# Does loop length change after anterior cruciate ligament reconstruction with adjustable loop cortical suspension device?: Observation of the hamstring graft completely filling the femoral tunnel

**DOI:** 10.1186/s40634-023-00629-5

**Published:** 2023-07-01

**Authors:** Kojiro Hyodo, Akihiro Kanamori, Ryunosuke Watanabe, Takeshi Ainoya, Naoya Kikuchi, Norihito Arai, Masashi Yamazaki

**Affiliations:** 1 Department of Orthopaedic Surgery, Faculty of Medicine, University of Tsukuba, 1-1-1Tennodai, Tsukuba, Ibaraki 305-8575 Japan; 2 Department of Orthopaedic Surgery, Tsukuba Memorial Hospital, 1187-299 Kaname, Tsukuba, Ibaraki 300-2622 Japan

**Keywords:** Anterior cruciate ligament reconstruction, Soft tissue graft, Adjustable loop cortical suspension device, Femoral cortical suspension device

## Abstract

**Purpose:**

The adjustable loop cortical suspension device (ALD) is a useful femoral fixation device in anterior cruciate ligament (ACL) reconstructions, but the possibility of loosening has been suggested. The purpose of this study was to evaluate the elongation of an adjustable loop and the position of the hamstring graft inside the femoral socket.

**Methods:**

The subjects were 33 patients who underwent ACL reconstruction with a hamstring tendon. The graft was fixed using ALD and completely filled the femoral socket. Magnetic resonance images were taken one week and one year after the operation. The loop length, femoral socket length, and graft length inside the socket were measured and statistically compared with the clinical outcomes.

**Results:**

The loop length one week after surgery was 18.9 ± 4.4 mm, and 1 year after surgery was 19.9 ± 4.5 mm (*P* < 0.001). The gap between the top of the graft and femoral socket was 0.9 ± 1.8 mm one week after surgery and 1.3 ± 1.7 mm one year after surgery (*P* = 0.259). At one week post-operation, a gap was found in nine patients (27.3%). The loop length and gap did not strongly correlate with clinical findings.

**Conclusion:**

ACL reconstruction using ALD showed a gap between the graft and femoral socket at the one week post-operation mark in 27.3% of participants. One year after the surgery, there were cases where the gap increased and/or decreased, but the elongation of the loop was 1 mm on average. Our findings suggest that ALD is clinically safe to use; however, has the possibility of initial loop elongation and non-uniform changes.

**Level of evidence:**

IV.

## Introduction

The cortical suspensory fixation device is widely used for femoral fixation in anterior cruciate ligament (ACL) reconstructions using the hamstring tendon. Conventionally, fixed loop cortical suspension devices (FLDs) have been used, and beneficial biomechanical and clinical results have been reported [8, 11, 17, 22]. However, there are some disadvantages, such as improper graft insertion due to incorrect measurement of femoral tunnel length and drilling length, and difficulty in use in short tunnels. Recently, a second-generation cortical suspension device has been developed. The adjustable loop cortical suspension device (ALD) has only one size and the insertion length of the graft tendon can be adjusted. The advantages are that it can be inserted into the bone socket to the maximum extent and the bungee effect [14] which is one of the factors that cause the enlargement of the tunnel is reduced. It is expected that the bungee effect will be reduced by completely filling the bone socket with the graft, which improves bone-tendon healing. Furthermore, it is easy to use even with a short tunnel, and it can be re-tensioned after fixation on the tibial side [3, 4, 21]. While clinical results are comparable to FLDs, biomechanical studies have warned that the adjustable loop may loosen and lengthen under cyclic loading [1, 3].

There are many studies of biomechanics about ALD, but most of these are in vitro evaluations of ALDs alone or on the knees of cadaveric and animal studies. To date, the in vivo condition of a tendon graft completely filling the bone tunnel using ALD remains unclear. Therefore, the purpose of this study was to evaluate the loop length changes and the position of the graft which is completely filled in the femoral socket after ACL reconstruction with ALD by magnetic resonance imaging (MRI) and compare them with clinical outcomes. Since previous biomechanical studies have shown that the ALD tends to become loosened with cyclic loading, we hypothesize that the loop would exhibit elongation in vivo, and the tendon graft would shift with it. However, the results were not related to clinical results.

## Materials and methods

### Patient recruitment

This study was a retrospective study. The subjects were 33 patients who underwent ACL reconstruction using an ALD from January 2016 to January 2018 at our hospital and related facilities. Inclusion criteria were primary anatomical single-bundle ACL reconstructions, with hamstring autograft. In all cases, the tendon was fixed to the femur using an ALD, and the button was in contact with the femur on a plain radiograph immediately after the operation. Those cases using an FLD (3 cases), double-bundle reconstruction (5 cases), bone patellar tendon-bone graft (BTB) (1 case) and quadriceps tendon-bone graft (2 cases), and revision ACL reconstructions (2 cases) were excluded. Table 1 shows the details of the patients. All patients provided informed consent. This retrospective study was approved by the institutional review board.

**Table 1:** Patient characteristics

	***n* = 33**
Age (years)	21.5 ± 10.8 [14–60]
Sex (male/female)	10/23
Side (right/left)	13/20
BMI (kg/m^2^)	23.3 ± 3.7
MRI imaging interval (m)	12.3 ± 2.2
Meniscal injury, n (%)	18 (54.5)
Medial	10 (30.3)
Lateral	11 (33.3)
Partial meniscectomy, n (%)	4 (12.1)
Medial	3 (9.1)
Lateral	2 (6.1)
Meniscal repair, n (%)	6 (18.2)
Medial	4 (12.1)
Lateral	2 (6.1)

Data are expressed as average ± standard deviation

*BMI* Body mass index, *MRI* Magnetic resonance imaging

### Surgery procedure

All operations were performed by senior coauthors and the procedure was the same in all cases. The semitendinosus tendon (ST) was harvested and quadrupled, for a graft diameter of 7–9 mm and a minimum length of 55 mm. When the thickness was not sufficient, the gracilis tendon was also harvested, combined with the ST to make a six-fold graft, and pretensioned. The femoral bone tunnel was drilled using an outside-in technique with an Antero-Lateral Entry Femoral Aimer (Smith & Nephew Endoscopy, Andover, MA) inserted with a guide pin into the anatomical centre of the ACL attachment behind the ‘resident’s ridge’. Using a Flip Cutter (Arthrex, Naples, FL) of the same diameter as the graft, 12–20-mm femoral socket was prepared according to the prepared graft length. For the tibial tunnel, a guide pin was inserted into the centre of the ACL attachment of the tibial stump using a Director Tibial Guide (Smith & Nephew Endoscopy, Andover, MA), and overdrilled with the same diameter as the tendon graft. The femoral side was fixed with TightRope RT (Arthrex, Naples, FL). After graft passage and confirming the button was flipped by intraoperative fluoroscopy, the graft tendon was completely inserted in the socket. After applying preconditioning for 60 s at 100 N and repetitive knee flexion–extension to fully remove the creep, the graft was fixed at 20°of knee flexion with a Double Spike Plate System (Smith & Nephew Endoscopy, Andover, MA) at 20 N by ligament tensioners (Smith & Nephew Endoscopy, Andover, MA). Meniscal resection, sutures, and cartilage procedures were added according to the arthroscopic findings.

### Postoperative therapy

All patients were rehabilitated according to the rehabilitation protocol performed at our hospital. The knee joint was placed in a brace for one week after surgery. Subsequently, joint range of motion exercise was started, and the range of motion was gradually increased weekly. After six weeks, there was no restriction. Partial weight bearing was started one week after the operation, and full weight bearing was taken in three weeks. When concomitant meniscus repair was performed, weight bearing and range of motion exercise were delayed for one-two weeks. Approximately three months after the operation, the patients were allowed to start running. Jump exercise was allowed at four months and participants returned to sports eight to 12 months after the operation.

### MRI Evaluation

**Fig. 1:**
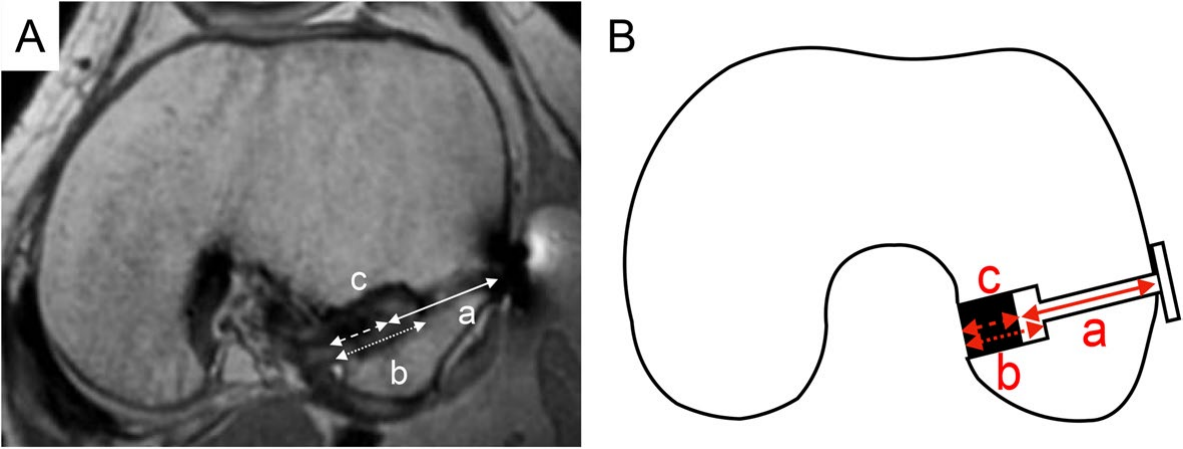
**A** Measuring method of magnetic resonance imaging with multiplanar reconstruction. A plane directly parallel to the femoral tunnel is used for evaluation. a: loop length (solid arrow); b: socket length (fine dotted arrow); c: graft length inside socket (coarse dotted arrow). **B** Schema of measuring method of magnetic resonance imaging with multiplanar reconstruction

We had taken MRI with the following protocol in a past study of bone tunnel enlargement. One week and one year after the operation, the MRI (Philips Ingenia 3.0 T or Achieva 1.5 T, Philips Healthcare, Best, The Netherlands) examination of axial 3D proton-density-weighted imaging sequences was performed before knee range of motion exercise for all cases, and the angle of knee flexion was fixed at 10°. The following parameters were used for imaging acquisition: repetition time = 1500 ms, echo time 39 ms, field of view = 130 mm, matrix = 216 × 216, and slice thickness = 0.6 mm. Multiplanar reconstruction was then performed using the Synapse VINCENT medical imaging system (Fujifilm Medical, Tokyo, Japan). First, an axis parallel to the femoral tunnel was created and adjusted to maximize the diameter of the femoral tunnel, femoral socket, and graft inside the socket. The distance from the lateral wall of the femur to the graft tendon (loop length) (a), the distance from the femoral tunnel aperture to the femoral socket (socket length) (b), and the distance from the femoral tunnel aperture to the graft tendon in the socket (graft length inside socket) (c) were measured (Fig. 1).

The change in the ALD loop length and the change in the gap between the top of the graft and the top of the femoral socket (b-c) were calculated. The measurements were performed by a single author, who was an orthopaedic surgeon with 12-years of experience.

### Clinical evaluation

Clinical results were evaluated before, and minimum two years after surgery. A Lachman test was graded as negative (≤ 2 mm), trace (3–5 mm), or positive (≥ 6 mm). Pivot-shift test was graded as equal, glide, clunk, or gross. Furthermore, Lysholm score, Tegner activity scale, and side-to-side difference measurements of a KT-1000 arthrometer (MEDmetric, San Diego, CA) performed at maximal manual forces were evaluated.

### Statistical analysis

Data were expressed as mean ± standard error. One-week and one-year measurements were compared using a paired t-test. Spearman correlation tests were performed between the Lachman and pivot-shift tests and variations in loop length and gaps. Pearson correlation tests were performed between the Tegner activity scale scores, Lysholm scores, KT-1000 measurements, and variations in loop length and gaps. Intra-observer and interobserver reliabilities of socket length and graft length inside socket were assessed using the intraclass correlation coefficient (ICC). The measurements were performed twice with an interval at least eight weeks between measurements to minimize the memory effect. A co-author also performed measurements to assess ICC. The ICC value for the interobserver reliability was 0.62–0.83. The ICC value for the intra-observer reliability was 0.87–0.93. A post-hoc power analysis was performed using alfa = 0.05 and N (number of cases) = 33; the power was over 0.9. For all statistical evaluations SPSS version 26.0 (IBM, Tokyo, Japan) was used, and significance was assumed at *P* < 0.05.

## Results

The loop length one week after surgery was 18.9 ± 4.4 mm, and one year after surgery was 19.9 ± 4.5 mm, and the change was 1.0 ± 1.4 mm (*P* < 0.001). The gap between the top of the graft and the top of the femoral socket was 0.9 ± 1.8 mm one week after surgery and 1.3 ± 1.7 mm one year after surgery, and the change was 0.4 ± 2.1 mm (*P* = 0.259) (Table 2).

**Table 2:** Results of MRI measurement

	**1 week after surgery**	**1 year after surgery**	***P* value**
Loop length (mm)	18.9 ± 4.4	19.9 ± 4.5	*P* < 0.001
Femoral socket length (mm)	13.8 ± 2.4	12.7 ± 1.9	*P* = 0.003
Graft length inside socket (mm)	13.0 ± 2.5	11.3 ± 2.3	*P* < 0.001
Tunnel-graft gap (mm)	0.9 ± 1.8	1.3 ± 1.7	*P* = 0.259

**Fig. 2:**
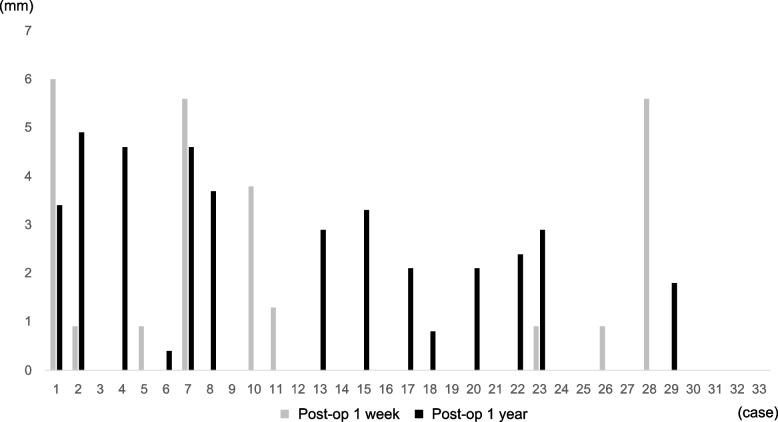
Details of change in tunnel-graft gap. Some cases with no data mean that the tunnel-graft gap does not exist

**Fig. 3:**
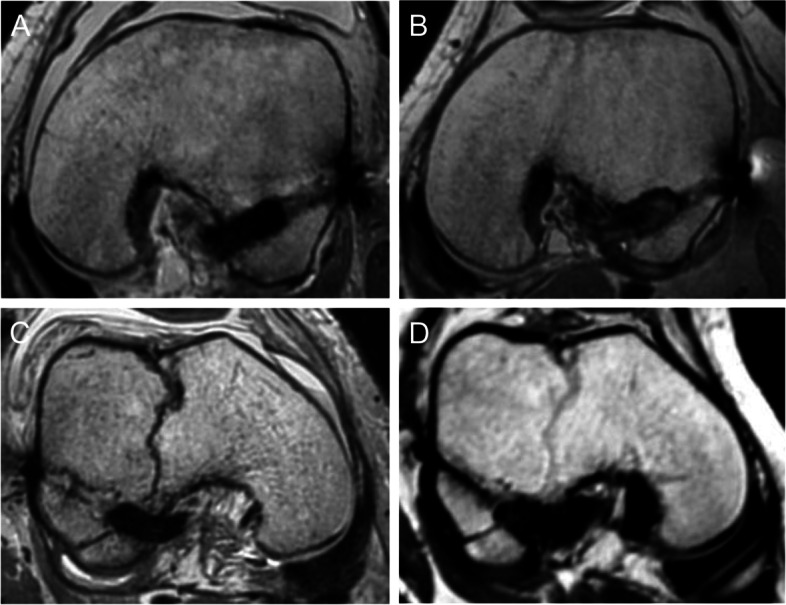
Magnetic resonance images. **A** An image of the left knee of a 43-year-old female one week after surgery shows that the hamstring graft was completely inserted in the socket. **B** An image of the left knee of a 43-year-old female one year after surgery (same case of A) shows that there is a gap between the top of the femoral socket and the graft. **C** An image of the right knee of a 14-year-old female one week after surgery shows that the hamstring graft is not completely inserted. **D** An image of the right knee of a 14-year-old female one year after surgery (same case of C) shows that there is no gap between the top of the femoral socket and the graft

At one week postoperatively, a gap was found in nine cases (27.3%), and a gap of 3 mm or more was found in four cases (12.1%). One year after surgery, the gap increased in 12 cases (36.4%) and decreased in seven cases (21.2%). There were four cases (12.1%) in which the gap increased by 3 mm or more and two cases (6.1%) in which the gap decreased by 3 mm or more (Fig. 2). Figure 3 shows an example of the MRI images. In cases with decreased the gap, gap was replaced by bone formation in all cases.

Regarding clinical evaluation, the range of motion of the knee joint was not different from that of the healthy side two years after surgery, and the Lachman test was negative in all cases. In the pivot-shift test, equal was recorded in 25 cases (75.8%), glide in eight cases (24.2%), and clunk and gross were not observed. The KT-1000 side-to-side difference measurement was 6.0 ± 2.1 mm preoperatively and 0.2 ± 1.1 mm postoperatively. The Tegnar activity scale was 7.0 ± 1.1 before surgery and 6.9 ± 1.1 after surgery. The Lysholm score was 82.9 ± 10.5 before surgery and 99.6 ± 1.4 after surgery (Table 3).

**Table 3:** Clinical outcomes

	**Pre-operation**	**Post-operation**
Lachman test, n (%)		
Negative	0 (0)	33 (100)
Trace	0 (0)	0 (0)
Positive	33 (100)	0 (0)
Pivot-shift test, n (%)		
Equal	6 (18.2)	25 (75.8)
Glide	23 (69.7)	8 (24.2)
Clunk	4 (12.1)	0 (0)
Gross	0 (0)	0 (0)
KT-1000 side-to-side difference (mm)	6.0 ± 2.1	0.2 ± 1.1
Tegner activity score	7.0 ± 1.1	6.9 ± 1.1
Lysholm score	82.9 ± 10.5	99.6 ± 1.4

The loop length change was not strongly correlate with the Lysholm score (*r* = -0.54, *P* = 0.001), mean KT-1000 side-to-side difference measurement (*r* = -0.18, *P* = 0.419), Lachman test (*r* = 0), pivot-shift test (*r* = -0.09, *P* = 0.621), or Tegnar activity scale (*r* = -0.09, *P* = 0.606). Change of tunnel-graft gap also did not strongly correlate with the Lysholm score (*r* = -0.07, *P* = 0.698), mean KT-1000 side-to-side difference measurement (*r* = -0.35, *P* = 0.106), Lachman test (*r* = 0), Pivot-shift test (*r* = -0.07, *P* = 0.717), or Tegnar activity scale (*r* = -0.23, *P* = 0.203).

## Discussion

The most important finding of this study was ALD showed non-uniform deformation in vivo after ACL reconstruction. This study evaluated the elongation of an adjustable loop and the position of the hamstring graft, which completely filled in the femoral socket in ACL reconstruction by MRI evaluation. Recently, ALDs have been used because of its technical easiness, but there are no certain conclusions regarding its mechanical properties. In our study, 9 cases (27%) had a gap between the graft and the socket one week after surgery. In our procedure, after confirming the flip of the Tightrope RT button, the loop was tensioned until it did not shrink further, and the graft was fully filled in the bone socket. The presence of a gap between the graft and the socket at one week after the operation indicates that the adjustable loop had already lengthened even after the early postoperative period.

Choi et al. reported the presence of a gap between the graft and the femoral socket on MRI images taken the day after an ACL reconstruction using, as in the present study, an ALD [6, 7]. They pointed out early postoperative gaps, and it is possible that the loop loosened. In a biomechanical cyclic loading test, there is a risk of adjustable loop plastic deformation. Therefore, we performed preconditioning of 100 N to fix the graft tendon; however, there was limited time to apply tension and the creep may not completely disappear. This may be the cause of the gaps within our procedures. In some studies, to avoid this phenomenon, re-tensioning to tighten the loop further after fixing the graft on the tibial side is recommended [7, 13]. However, this procedure may increase the initial tension applied at tibial fixation, and which may result overconstrained knee. Therefore, we did not include this in our procedures. It is up to the surgeon to allow a 1 mm gap or to choose a hyper-tensioned graft.

In this study, at one year after the operation, the gap increased in 12 cases (36%), while the gap decreased in seven cases (21%). This suggests that the mechanical properties of an ALD may affect the dynamics of the tendon in the bone socket, even long after surgery. There have been few reports on gaps in the femoral socket with cortical suspension devices using MRI. Ahn et al. reported about 10% of cases with a tunnel-graft gap at six months after surgery in both FLD and ALD, but this gap was not related to the clinical results [2]. Choi et al. pointed out that the factor contributing to the gap increased six months after surgery. They reported that the loop loosened, possibly due to the rehabilitation load [7]. In our study, there were cases in which the gap increased one year after the operation. This is because the ALD loosened during plastic deformation by cyclic loads [1]. However, Iuchi et al. reported that the ALD showed a smaller elongation of the loop with increases in the lower force limit and with lower cyclic loading speeds, and the postoperative load was different in each case [15]. This result should be taken into consideration when applying the load up to three months after surgery, which is considered to complete the healing of the graft tendon in the bone tunnel. Load up during this period should be considered more carefully. In this study, the gap became shorter in six cases, in contrast to the report by Choi et al. [7]. When the MRI images of all six cases were evaluated, the gap appeared to be filled with bone. This phenomenon was only observed in our study. This might be due to the difference in the measurement method and accuracy. It is necessary to study further how bone formation in the socket progresses over a long period of time.

To date, several studies have reported the biomechanical properties of ALDs. Several papers have reported that the maximum displacement of loop length after cyclic loading is larger in ALD compare to FLD [3, 5, 10, 17, 22]. Ahmad et al. showed that the ALD loop undergoes plastic deformation due to cyclic loading, and these devices show biomechanical inferiority and demonstrate heterogeneity of fixation properties [1]. In addition, regarding ultimate load to failure, there are many reports that ALDs have a weaker breaking strength than FLDs [9, 16, 20, 22]. However, there are still many unclear factors regarding the elongation of the adjustable loop in vivo. Kusano et al. evaluated the loop length change of the ALD by computed tomography after ACL reconstruction using BTB, but reported that the elongation was only 0.04 mm from one to 12 weeks after surgery [18]. This study also evaluated the change in loop length. The loop length increased by 1 mm on average from one week after surgery to one year after surgery. Since BTB and hamstring have different healing properties [19], a simple comparison cannot be made. However, none of the loops showed catastrophic elongation, and gap formation and loop length were not associated with clinical outcome. Given the technical benefits of ALDs, an average 1-mm elongation of loop may not be a concern. To date, there have been some reports that there is no significant difference between ALD and FLD in clinical results, such as Lysholm score, IKDC score, KT-1000 arthrometer laxity, Lachman test, and pivot-shift test [2, 4, 12, 19, 23].

This study has several limitations. The first is the potential for measurement accuracy. Although there was no problem in the measurements one week after the operation, there were some cases in which it was difficult to evaluate the bone socket and the tendon graft one year after the operation, depending on the degree of enlargement of the femoral tunnel and ‘ligamentization’ of the graft. Intra-rater and inter-rater errors were evaluated using reconstructed images from high-resolution MRI scans and were considered acceptable. Second, the initial measurement of loop length was performed one week after the operation.

There might have been loosening during this period. However, since the knee has been fixed for one week and the load has been limited, this bias was mitigated as much as possible. Third, there was no control group. Since the in vitro biomechanical superiority of an FLD is clear, it is considered that the elongation of the loop is less than that of ALD. We also did not evaluate the enlargement of the femoral tunnel, which is considered one of the advantages of ALDs. Since it is clinically relevant to evaluate femoral tunnel enlargement by ALDs, it is necessary to evaluate this in future studies.

## Conclusion

ACL reconstructions using an ALD in which the hamstring tendon fully filled the femoral socket showed a gap between the top of the graft and the top of the femoral socket in 27.3% of cases one week after the operation, and one year after the surgery, there were cases where the gap increased and decreased. In this study, ALD showed non-uniform deformation in vivo, but it is clinically safe to use if it is used in consideration of the possibility of initial loop elongation.

## Abbreviations

ACL: Anterior cruciate ligament
ALD: Adjustable loop cortical suspension device
BTB: Bone patellar tendon-bone
FLD: Fixed loop cortical suspension device
ICC: Intraclass correlation coefficient
MRI: Magnetic resonance imaging
ST: Semitendinosus tendon
